# Perspectives on Battling COVID-19 in Countries of Latin America and the Caribbean

**DOI:** 10.4269/ajtmh.20-0571

**Published:** 2020-06-09

**Authors:** Jon Kim Andrus, Tracy Evans-Gilbert, Jose Ignacio Santos, Maria G. Guzman, Philip J. Rosenthal, Cristiana Toscano, Maria Teresa Valenzuela, Marilda Siqueira, Carissa Etienne, Joel G. Breman

**Affiliations:** 1 Department of Global Health, Milken Institute of Public Health, George Washington University, Washington, District of Columbia;; 2 Division of Vaccines and Immunization, Center for Global Health, Colorado School of Public Health, Aurora, Colorado;; 3 Department of Child and Adolescent Health, University of the West Indies, Kingston, Jamaica;; 4 Global Health Program, Cornwall Regional Hospital, West Virginia University School of Medicine, Jamaica;; 5 General Health Council of Mexico, Mexico City, Mexico;; 6 Center for Research, Diagnostic and Reference, Tropical Medicine Institute Pedro Kouri, Havana, Cuba;; 7 Department of Medicine, University of California San Francisco, San Francisco, California;; 8 Department of Collective Health, Institute of Tropical Medicine and Hygiene, Federal University of Goiás, Goiânia, Brazil;; 9 Faculty of Medicine, Special Advisor to the Ministry of Health for COVID-19, University of the Andes, Santiago, Chile;; 10 Respiratory and Measles Viruses Laboratory/SARS CoV 2 Reference, Laboratory, MoH, WHO, FIOCRUZ, Rio de Janeiro, Brazil;; 11 Pan American Health Organization, Washington, District of Columbia;; 12 American Society of Tropical Medicine and Hygiene, Arlington, Virginia

## INTRODUCTION

The first case of COVID-19 reported in Latin America occurred in São Paolo, Brazil, on February 25, 2020, in a 61-year-old man who had recently returned from the Lombardy region in Italy. The first cases in the Caribbean region were reported on March 1 in St Martin in a couple who returned from France and in the Dominican Republic in a 61-year-old man visiting from Italy.^1,2^ By mid-March, there was a substantial surge in cases, resulting in nearly every country in Latin America and the Caribbean (LAC) reporting COVID-19 (Table 1). These data are reported to the Pan American Health Organization (PAHO) regional office from national ministries of health and as such may have inherent weaknesses of timing, completeness, and accuracy. However, they do provide insight on trends and hotspots in the region.

**Table 1:** Data on COVID-19 from selected countries in Latin America and the Caribbean

Country	Total cases	Total deaths	Recovered	Population (in 1,000’s)	Diagnostic tests performed per 1 million population
Chile	82,289	841	33,540	19,116	25,188.4
Colombia	23,003	776	5,511	50,883	5,090.6
Peru	129,751	3,788	52,906	32,972	25,663.8
Argentina	13,288	492	4,349	45,196	2,908.6
Brazil	391,222	24,512	158,593	212,559	3,509.9
Uruguay	789	22	638	3,474	11,059.1
Ecuador	38,103	3,275	18,425	17,643	6,209.1
Panama	11,447	313	6,379	4,315	13,943.4
Venezuela	1,245	11	302	28,436	27,848.9
Paraguay	884	11	392	7,133	3,567.0
Belize	18	2	16	398	3,746.0
Costa Rica	956	10	634	5,094	4,676.1
Honduras	4,401	188	493	9,905	1,232.9
Guatemala	3,954	63	289	17,916	1,476.4
El Salvador	2,109	37	873	6,486	11,309.6
Mexico	74,560	8,134	52,219	128,933	1,736.8
Dominican Republic	15,723	474	8,790	10,837	6,149.8
Haiti	1,174	33	22	11,403	244.4
Jamaica	564	9	211	2,961	3,485.7
Trinidad and Tobago	116	8	108	1,399	2,108.1
Antigua and Barbuda	25	3	14	98	1,900.6
Saint Vincent and the Grenadines	25	0	14	111	1,896.4
Saint Lucia	18	0	18	184	4,937.1
Dominica	16	0	16	72	6,045.4
Cuba	1,974	82	1,724	11,327	8,295.9

Data source: Reported from countries to Pan American Health Organization (https://ais.paho.org/phip/viz/COVID19Table.asp) as of May 27, 2020.

On recognition of importations from Europe, most countries and territories curtailed international arrivals and enacted severe public health and societal measures. Supported by the PAHO, countries of LAC organized quickly to set up response procedures.^3^ However, as of this writing, transmission continues to increase in several countries of LAC with less stringent measures. Brazil now stands to become the newest epicenter.

As experienced elsewhere in the world, in hotspots where transmission has been most intense, such as in Guayas Province in Ecuador, health systems have been stretched to the limits, especially with regard to intensive care unit beds and ventilators (Table 2). Countries have different levels of resiliency in their health systems. Where transmission has been limited, health systems have coped, probably helped by a younger population demographic in LAC compared with North America and Europe (median age is 31.9 years in LAC, 38.3 years in the United States, and 47.3 years in Italy). Although variably effective from a public health standpoint, the enacted measures have impacted all national economies, including those of the smaller, tourism-dependent Caribbean island countries.

**Table 2:** COVID-19–related hospital admissions, ICU admissions, ventilated patients, and hospital beds in selected countries of Latin America and the Caribbean*

Countries	Hospital admissions	Hospital beds/1,000 population	Intensive care unit admissions	Patients ventilated
Chile	6,168	2	1,905	715
Colombia	284	1.7	270	0
Argentina	5,739	5	305	251
Ecuador	567	1.5	458	11
Paraguay	5	1.3	3	2
El Salvador	43	1.3	1	0
Costa Rica	76	1.2	30	2
Belize	4	1.3	3	0
Honduras	5	0.7	1	1
Mexico	66,770	1.5	2,517	2,517
Dominican Republic	2,927	1.6	304	304
British Virgin Islands	2	No data	1	1
Jamaica	41	1.7	0	0
Barbados	3	5.8	1	0
Antigua and Barbuda	3	3.8	0	0
Saint Kitts and Nevis	2	2.3	0	0

Data source: http://data.worldbank.org y https://data.oecd.org/and https://ais.paho.org/phip/viz/COVID-19EpiDashboard.asp

* Based on available data as of May 28, 2020.

Daily COVID-19 knowledge continues to grow with regard to disease dynamics and, most appropriate, life-saving responses. As the situation evolves, countries are struggling to “fly the plane as it is being built.” But, as with previous infectious disease epidemics, critical guiding principles are grounded in sound public health practice, including community engagement, surveillance anchored in laboratory confirmation, rapid public health response after the detection of first cases, technical and operational oversight, effective case management, access to information and technologies, effective communication among countries, and strategic leadership invested in community engagement and ownership.^4^ Countries remain vulnerable to public health emergencies, as COVID-19 shows. Unlike the situation in many South Asian countries, the experience gained from earlier threats over the years has waned over time, compounded by misguided or absent political commitment. However, technical teams in most LAC countries have worked to strengthen and sustain surveillance.

Many LAC countries acknowledge the importance of case identification and isolation, contact tracing, and quarantine, while continuing to implement physical distancing and hygienic measures. But, the extent to which countries embrace these guiding principles varies substantially across, and within, countries of LAC. This is most obvious in Brazil, where political issues have endangered evidence-based public health action. We wish to highlight four critical areas of concern relevant to countries of LAC that should be prioritized to reduce mortality and eventually suppress transmission: surveillance and laboratory confirmation, response capacity and evidence, access to medicines and vaccines, and leadership and coordination.

## SURVEILLANCE AND LABORATORY CONFIRMATION

Latin America and the Caribbean countries developed strategic plans for combatting a potential H5N1 influenza emergence in 2004, focusing on surveillance capacity.^5^ This preparedness planning served countries well when H1N1 influenza emerged from Mexico in 2009, becoming a pandemic of global concern. Countries mobilized to implement prevention and control measures recommended by PAHO, which convened regular meetings to share information and coordinate efforts. The U.S. CDC played a fundamental role in the development and distribution of laboratory tests and supplies in addition to ongoing efforts of international capacity building in field epidemiology.

After the 2009 influenza pandemic, PAHO continued to work with countries in consolidating a regional system, part of the global network, for the surveillance of influenza and other respiratory viruses. This global network is backstopped by a network of national reference laboratories, most of which are formally recognized as National Influenza Centers (NIC) within the 68-year-old Global Influenza Surveillance and Response System.

When COVID-19 emerged in LAC in February 2020, the region quickly leveraged existing network capacity so that by February 24, all LAC countries could establish in-country testing or had access to a subregional laboratory that could test. In particular, PAHO selected a molecular testing protocol from Hospital Charite, Berlin, a reputed laboratory in Germany; acquired and distributed as much as possible the necessary supplies; partnered with the NIC in Brazil and Mexico to offer hands-on training to all countries; and carried out missions to support in-country proficiency. Pan American Health Organization was able to conduct this work using a global collaborative approach. However, testing supplies and equipment provided by PAHO fell far short of demand. A remaining challenge is for countries to scale up testing capacities, for instance, by decentralization at the subnational level so that all COVID-19 cases can be detected and contacts traced. Brazil also quickly developed and is producing laboratory tests for country use. But, production of kits is restricted by dependence on external reagents. This restriction also affects supplies required for the sampling of patients, and, as at the regional level, available supplies fall far short of local needs.

Pan American Health Organization works closely with countries to integrate COVID-19 into the existing sentinel-based surveillance of severe acute respiratory infections (hospitalized patients) and influenza-like illness (ambulatory patients) already in place as part of long-standing global influenza surveillance and more recently established surveillance for severe respiratory diseases since SARS and H1N1 influenza. Such an integration of approaches will help ensure long-term sustainable COVID-19 surveillance for a virus clearly expected to be around a while. This is also being done for influenza and syncytial respiratory viruses.

## RESPONSE CAPACITY AND EVIDENCE

The COVID-19 pandemic is highly dynamic. COVID-19 data of today do not represent what the situation will be tomorrow or next week. Infections occurring today will require hospital services for the next 1–2 weeks. Deaths generally occur 2–3 weeks after symptom onset. Decisions made today on return to work and to open economies must be grounded in the evidence but more importantly effectively monitored so that rapid adjustments can be implemented to prevent unwarranted deaths.

In the absence of vaccines, therapeutics, and sufficient critical care capacity, with case counts doubling every few days, most LAC countries promptly implemented recognized social distancing measures. But the extent and impact of these mitigating strategies and other measures has been highly variable across and within countries. In the Caribbean, many countries implemented strict lockdown policies early in the epidemic that likely contributed to comparatively lower death rates. In Jamaica, for example, cases were identified through contact investigation and sentinel surveillance and quarantined in government isolation facilities. Where clusters were identified, affected communities were placed under lockdown for a 14-day period to contain the spread.

The Caribbean Public Health Agency activated its incident management team and coordinated the regional preparedness and response by mid-January. By February, many Caribbean countries had initiated efforts to reduce exposure of vulnerable populations with social distancing, personal measures, and use of masks. Ongoing surveillance efforts to detect, isolate, and treat patients and their contacts were reinforced. Most Caribbean countries adopted a whole of government, whole of society approach, which included tight cooperation across all government sectors, as well as buy-in from communities and private sectors. Strict lockdown could not have been implemented in the Caribbean without such cooperation, which to date has been associated with lower mortality rates from COVID-19 than that of other countries in the region. Cuba has provided support with trained medical personnel to countries of the Caribbean and other parts of the world, where human resources are limited.

Effective supply chain logistics requires timely data on the status of critical response items to match scarce global availability (Table 2). This has been an important component of emergency response preparedness plans in the past. Comprehensive reviews of national personal protective equipment requirements and laboratory supplies and equipment, as well as real-time understanding of the availability of critical care items, such as ventilators, remain critically important to guide corrective action. Such data can also be used to advocate globally for more readily available, robust supply chains.

Recognizing public hospitals were soon to reach capacity limits; on March 19, 2020, the General Health Council of Mexico negotiated an agreement with 143 certified private hospitals from the National Association of Private Hospitals and the Consortium of Private Hospitals. The private hospitals agreed to convert themselves into COVID hospitals and also to treat non-COVID patients when government hospitals had exceeded capacity. This agreement, the first of its kind, added an additional > 1,700 hospital beds to Mexico’s public hospital infrastructure.^6^

Effective, real-time surveillance in Cuba requires daily reporting of all suspect COVID-19 cases by family doctors, medical students, and nurses at all points of service in the national primary healthcare system.^7^ Although an impact evaluation was not conducted, this network of providers implemented standardized treatment protocols that are believed to have led to reduced serious illness.

However, many areas throughout LAC have no information, or substantial gaps in information. Epidemiologic details are often lacking because of challenges in case ascertainment bias and the lack of testing mentioned previously.

In Brazil, early social distancing measures were implemented in most of its 27 states. On February 6, 2020, Brazil enacted a law with provisions for strict social distancing measures to address the COVID-19 epidemic. By the second week of March, states began to stagger the implementation of these measures, according to the timing of the epidemic locally. In São Paulo, the most populous state in the country and where the epidemic was first identified, rigorous quarantine measures were implemented during the third week of March. Keeping these strict measures in place has proven challenging in most states, and even more so, given challenges to some public health efforts from the federal government.^8^ Unfortunately, the federal government downplayed the seriousness of the epidemic. That and the staggering of efforts leading to delays have contributed Brazil’s emergence as an epicenter.

As in other parts of the world, the epidemic strikes the most vulnerable in LAC, including indigenous populations of the Amazon basin. Amazonian Iquitos in Peru does not have personal protective equipment, and even oxygen, let alone supportive care. Dengue and other tropical infectious diseases are ongoing. Similar challenges have occurred in Brazil and most likely throughout the Amazon basin.

## ACCESS TO MEDICINES AND VACCINES

Outside of hotspots, an estimated 99% of the LAC population remains susceptible to COVID-19. Notwithstanding the uncertainty and challenges in developing, producing, and delivering at scale a safe an efficacious vaccine, immunization is today the only tangible prospect for lasting control of COVID-19.

Mexico has been particularly forthright in raising its access concerns to the global community. On March 26, the G-20 held a virtual summit focusing on the COVID-19 pandemic. In that meeting, the president of Mexico proposed intervention of the United Nations to ensure that all countries have equal access to medicines and medical equipment which, because of the ongoing pandemic, are being hoarded by those countries with an economic advantage. He indicated that the United Nations should intervene to avoid economic speculation and inappropriate profit gains stemming from the sale and purchase of medicines and medical equipment. On April 20, the Mexican resolution, endorsed by 179 countries as cosponsors, was adopted by consensus as General Assembly resolution 74/274, entitled “International cooperation to ensure global access to medicines, vaccines, and medical equipment to face COVID-19.”^9^

As there are no currently approved drugs for the treatment of COVID-19, specific medical interventions in most countries have been mainly supportive. Lessons learned from clinicians in countries with large outbreaks regarding ventilation, managing coagulopathies, and other clinical aspects of COVID-19 have been useful. Updates of clinical trials are frequently reviewed through PAHO and ministries of health. The WHO advises that repurposed drugs should be used only in the context of patient-consented, randomized clinical trials, such as the WHO Solidarity trial.

Pan American Health Organization plays a unique role in providing access to safe, affordable, and effective vaccines. Founded in 1979, the PAHO Revolving Fund does this using economies of scale to negotiate and purchase the vaccines needed by member countries, including more expensive newer vaccines.^10^ Equitable access to COVID-19 vaccines and fair allocation of resources to make vaccines available will continue to be a PAHO top priority. Vaccine producers also benefit from the Revolving Fund purchase mechanism, as evident with the H1N1 influenza experience, in which countries of LAC were second globally in the purchase of this vaccine.

Ultimately, the need to develop local capacity for the advancement of biomedical science is a key lesson learned to avoid dependence on wealthier countries for life-saving products that will likely be sequestered in countries of origin. Several LAC countries—Argentina, Brazil, Chile, Cuba, and Mexico, to name a few—have made remarkable progress in their biomedical research and development capacities.^11^ They also would benefit from collective action to counterbalance the sequestration of products in resource-rich countries.

## LEADERSHIP AND COORDINATION

Global leadership must be fit for purpose, moral based, and inclusive. It must also be based in sound and transparent communication and information sharing with society as a whole. COVID-19 is a global problem, which requires a global solution. In particular, coordinated efforts are needed to protect vulnerable populations.

Going forward, to paraphrase the prime minister of Barbados in her speech to the World Health Assembly in May, we cannot drop the ball on immunization services, prevention and control of chronic diseases, HIV infection, tuberculosis, and tropical infectious diseases. While facing the possibility of sustained COVID-19 transmission, political and technical leadership should expect, and be prepared for, large vaccine-preventable disease outbreaks, such as measles, in the near future, as many countries have not been able to keep up with essential immunization coverage during this crisis.

Countries must move quickly to build on lessons learned from this pandemic. Working collaboratively, mechanisms must be re-established and strengthened to prepare and respond to the inevitable, continued challenges of the years to come. To highlight the gravity of the situation in LAC, at the time of this writing, Haiti is emerging as another hotspot. The community of nations will again be challenged to help a vulnerable neighbor in need of support.
